# β-Hydroxybutyrate-induced mitochondrial DNA (mtDNA) release mediated innate inflammatory response in bovine mammary epithelial cells by inhibiting autophagy

**DOI:** 10.1186/s40104-024-01143-z

**Published:** 2025-02-01

**Authors:** Yihui Huo, Taiyu Shen, Tianyin Feng, Moli Li, Wanli Zhao, Juan J. Loor, Ben Aernouts, Androniki Psifidi, Chuang Xu

**Affiliations:** 1 College of Veterinary Medicine, China Agricultural University, Beijing, 100193 China; 2 Department of Animal Sciences, University of Illinois at Urbana-Champaign, Urbana, IL 61801 USA; 3 Department of Biosystems, Division of Animal and Human Health Engineering, KU Leuven, Campus Geel, Leuven, 2440 Belgium; 4 Department of Clinical Science and Services, Queen Mother Hospital for Animals, The Royal Veterinary College, London, UK

**Keywords:** Autophagy, Bovine mammary gland, Inflammation, Mitochondria DNA

## Abstract

**Background:**

In perinatal dairy cows, ketosis is a prevalent metabolic disorder that lowers milk output and performance. Mitochondrial dysfunction and chronic inflammation in mammary tissue are linked to elevated blood ketone levels, particularly β-hydroxybutyrate (BHB). Recent research has linked cytosolic mitochondrial DNA (mtDNA) with chronic aseptic inflammation by activating the cGAS-STING pathway during metabolic disorders, while autophagy activation effectively reverses this process. However, whether it is involved in mammary gland damage during ketosis is poorly understood. Therefore, this study aimed to explore the underlying mechanisms of mtDNA-induced inflammation under BHB stress and evaluate the potential therapeutic strategy of autophagy activation in mitigating this damage.

**Results:**

Our study found an increased cytoplasmic mtDNA abundance in mammary gland tissues of dairy cows with ketosis and bovine mammary epithelial cell line (MAC-T) subjected to BHB stress. Further investigations revealed the activation of the cGAS-STING pathway and inflammatory response, indicated by elevated levels of cGAS and STING, along with increased phosphorylation levels of TBK1, P65, and IκB, and higher transcript levels of pro-inflammatory factors (*IL-1B*, *IL-6*, and *TNF-α*) in both in vivo and in vitro experiments. Notably, STING inhibition via si-STING transfection reversed BHB-induced inflammation. Additionally, autophagy activation appeared to protect against BHB stress by facilitating the removal of cytoplasmic mtDNA and preventing cGAS-STING pathway-mediated inflammation.

**Conclusions:**

The findings illustrate that elevated BHB levels lead to the release of cytoplasmic mtDNA, which in turn activates the cGAS-STING pathway and triggers an inflammatory response in the mammary glands during hyperketonemia. Conversely, autophagy activation has been shown to alleviate this process by promoting cytoplasmic mtDNA degradation.

**Graphical Abstract:**

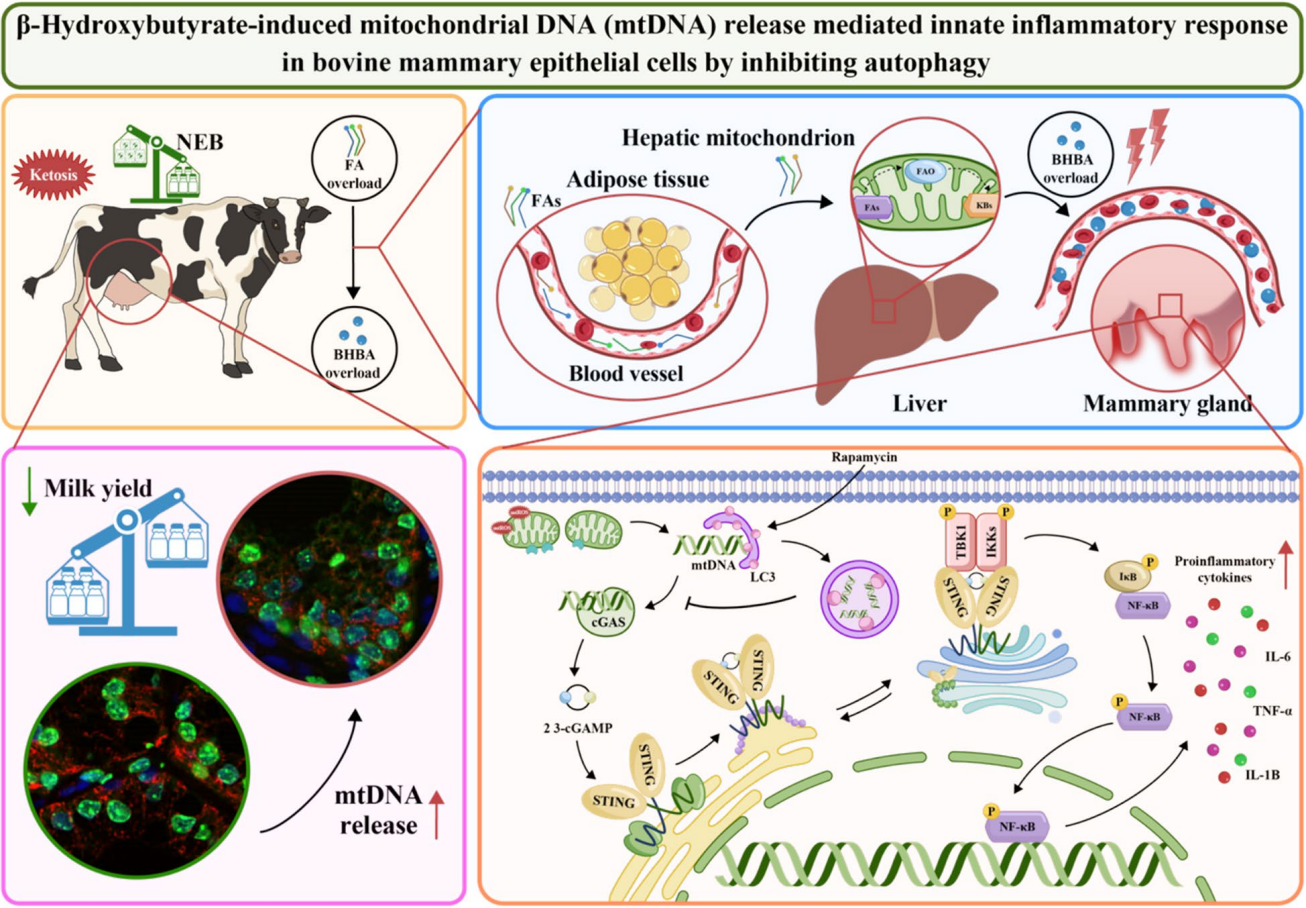

**Supplementary Information:**

The online version contains supplementary material available at 10.1186/s40104-024-01143-z.

## Introduction

Ketosis is a highly prevalent metabolic disorder in periparturient dairy cows, often leading to reduced milk yield and performance, with serious economic implications for farmers [1, 2]. It is also associated with an increased incidence of diseases such as mastitis [3, 4]. Although a moderate increase in blood β-hydroxybutyrate (BHB) induced by caloric restriction or ketogenic diets can be therapeutic in alleviating inflammatory diseases, BHB overload causes oxidative stress, triggers apoptosis, and mediates systemic inflammation in dairy cows [5–7]. Previous research also reported that typical inflammatory markers, including NF-κB signaling and NLRP3 inflammasome, were activated in the mammary glands of clinical ketotic cow [8]. These evidences imply that sterile inflammation might contribute to mammary gland dysfunction while the underlying mechanisms are not yet understood. To develop effective interventions, it is pressing to thoroughly elucidate these internal relationships, especially for important economic diseases such as intramammary inflammation.

Mitochondria are not only the primary energy producers of the cell but also crucial for regulating cellular endogenous inflammation [9]. During perinatal period, the coordination of mitochondrial bioenergetic and biosynthetic capacity are recognised as central to overcome metabolic challenges due to lactation [10]. However, studies revealed that mitochondrial activity and function are impaired in the mammary gland of dairy cows suffering from ketosis [11]. Abnormal mitochondrial homeostasis can lead to cytosolic mtDNA leakage, which promotes the activation of the cGAS-STING pathway and triggers a chronic sterile inflammatory response, facilitating the processes of aging, metabolic disorders, and autoimmune diseases [12–14]. Therefore, further investigation into mtDNA release under BHB stress is essential to understand its role in mammary gland inflammation during ketosis.

Autophagy is a refined self-degradation system mediating the removal of abnormal or damaged cellular contents, preventing the initiation of inflammation, and protecting cells from persistent stimulators of dangerous molecules [15]. As evidenced by the importance of autophagy in the proliferation and antioxidant capacity of mammary cells during lactation, the homeostasis of autophagy is crucial for cellular functionality and adaptability [16]. Autophagy activation also alleviates mastitis, partly by improving the blood-milk barrier integrity and reducing LPS-induced inflammatory damage [17]. Impaired autophagy promotes oxidative stress, whereas activated autophagy mitigates metabolic stress and enhances adaptive tolerance in mammary tissue during hyperketonemia [11, 18]. These findings suggest that enhancing autophagy may be a promising therapeutic strategy to prevent mammary gland injury during BHB stress.

Considering that hyperketonemia is a significant cause of mitochondrial dysfunction and systemic inflammation, the internal mechanism is still uncertain. In this study, we hypothesize that excess BHB induces mtDNA release, thereby activating inflammation through the cGAS-STING pathway in bovine mammary epithelial cells, which might be alleviated by improving autophagy activation.

## Materials and methods

### Animals and sample collection

In this study, a total of 101 Holstein cows (3–4 parities, within 21 days in milk) were selected from a 25,000-dairy farm located in Baoding, Hebei province, China. Cows were fed TMR (Table S1) 3 times per day and free access to water. Ten healthy cows (serum BHB ≤ 0.6 mmol/L) and 10 cows with clinical ketosis (serum BHB ≥ 3.0 mmol/L) without other comorbidities were selected based on a routine physical examination and serum BHB concentration analysis. All of the selected cows were moved to an experimental barn for subsequent sampling.

Blood samples were collected from caudal vein before morning feeding, kept at room temperature for 2 h, and centrifuged at 3,000 × *g* for 15 min at 4 °C to sepertated the serum. The serum concentration of NEFA and glucose were determined using biochemical analyzer (Roche Cobas 6000, Basel, Switzerland). For animal welfare reasons, we randomly selected 6 cows per group for subsequent biopsies. Mammary gland tissue samples were obtain from the right or left rear quarter using a Core Biopsy Instrument as described [19] under anesthesia. After biopsy, sterile gauze was applied to hemostasis by compression for 15 min and close the incision area with surgical staples. The collected mammary gland samples were washed with normal saline and stored at −80 °C for subsequent detection. The body condition scores (BCS) were assessed by a professional veterinarian in accordance with the earlier publications [20].

### Cell cultural and treatment

The bovine mammary epithelial cell line (MAC-T) was acquired from Shanghai Shunran Biology and cultured in complete medium: nutrient mixture F-12 medium (DMEM/F-12; 11330032, Gibco, USA) supplemented with 10% fetal bovine serum (FBS; FB15015, Clark, Cordova, Argentina) and 100 U/mL penicillin–streptomycin (15140122, Gibco, USA) as described in our previously study [21].

For in vitro experiment, MAC-T were seeded onto 6-well cell culture plates (1 × 10^6^ cells/mL) and cultured at 37 °C in 5% CO_2_ to ∼80% confluence. To establish BHB stress model, cells were serum-starved by incubating in DMEM/F-12 for 12 h. The stock solution of DL-β-hydroxybutyric acid sodium (BHB) (Cat. No. 150834, Sigma-Aldrich) was prepared as described previously and stored at −20 °C [22]. In accordance with hematology standards of clinically ketotic cows [6, 23, 24], MAC-T were stimulated with 2.4 mmol/L BHB for 0, 6, 12, 24, and 36 h, respectively. Then, MAC-T were stimulated with 0, 1.2, 2.4, and 3.6 mmol/L BHB in DMEM/F-12 for 24 h.

To explore the role of STING on the BHB-induced inflammation, si-STING or si-NC were transfected into MAC-T for 12 h using siRNA/miRNA Transfection Reagent (40806ES, Yeason, China) according to the manufacturer’s instruction. The siRNA sequences are shown in Table S2. To investigate the role of autophagy in BHB-induced mtDNA release, 100 nmol/L Rapamycin (Rapa, 53123-88-9, MedChemExpress, China) was applied to pretreat MAC-T for 2 h. Subsequently, MAC-T were incubated in DMEM/F-12 for 12 h and then stimulated with 2.4 mmol/L BHB for 24 h as mention above.

### Cytosolic mtDNA isolation and determination

The protocol for cytosolic mtDNA isolation was referred to previous publication [25]. Briefly, the treated cells were digested with pancreatic enzymes and centrifuged at 800 × *g* for 5 min. Subsequently, the cell pellet was resuspended with 1 mL PBS and divided into two tubes for isolating cytoplasmic DNA and whole-cell DNA, respectively. For cytoplasmic DNA extraction, the cell particles were incubated in 400 μL digitonin lysis buffer at 4 °C for 10 min to lyse plasma membranes while the mitochondrial membranes remained intact. The lysate was centrifuged at 17,000 × *g* for 10 min to remove cellular debris, and the supernatant was collected. A FastPure Cell/Tissue DNA Isolation Mini Kit (DC102, Vazyme, China) was applied to isolate DNA from the supernatant and whole cell pellet following the manufacturer’s instructions. The DNA concentration and the absorbance of 260/280 of each sample were assessed by a Thermo Scientific NanoDrop (Thermo Fisher Scientific, USA). Each sample was uniformly diluted into 2 ng/μL. The cytosolic mtDNA (*CYTB*, *ND1*, and *COXI*) abundance was analyzed by real-time PCR as a relative expression to nuclear DNA (TERT) of whole cell DNA. The primers used are presented in Table S3.

### Immunohistochemical (IHC) staining

Mammary tissues were fixed in 4% paraformaldehyde, embedded in parafin and cut into 4 μm thicknesses in accordance with previously described protocols [26]. Thereby, the mammary sections were incubated with primary antibodies against STING (1:200, bs-8335R, Bioss) overnight at 4 °C. HRP conjugated goat anti-rabbit IgG antibodies (1:200, CW0103S, CWBIO, Beijing, China) and DAB Horseradish Peroxidase Color Development Kit (P0202, Beyotime, China) were applied to display color. A light microscope (DM500, Leica, Germany) was applied to section observation, and ImageJ was applied to analyse the integrated option density (IOD) to intuitively observed the expression of STING in mammary tissues.

### Immunofluorescence (IF) staining

Cytokeratin 18 (CK18) Polyclonal antibody (1:200, 10830-1-AP, Proteintech) was used to verify the cell type. To observe the intracellular sublocalization of mtDNA in mammary tissues and MAC-T, Mito-Tracker Deep Red FM (C1032, Beyotime, China), COXIV Polyclonal antibody (1:200, 11242-1-AP, Proteintech, USA), and anti-DNA mouse monoclonal (1:200, 690014S, Progen, Germany) were applied to specifically label mitochondria and dsDNA.

### Monodansylcadaverine (MDC) staining

MDC is a specific dye for autophagic vacuole. To observe the formation of autophagosomes, cells were incubated with MDC staining reagent (KGA2402-100, KeyGEN BioTECH, Jiangsu, China) for 30 min in dark condition and observed with a confocal microscope (TCS SP8, Leica, Germany).

### Adenovirus transfections

The GFP-RFP-LC3 system is a widely used dual-marker tool for evaluating autophagy, distinguishing autophagosomes (yellow puncta) from autolysosomes (red puncta) based on the pH sensitivity of GFP fluorescence, allowing for a clear observation of changes in autophagic flux. To detect autophagic activity of MAC-T under BHB stress, mRFP-GFP-LC3 was transfected into MAC-T at an MOI of 50 for 6 h in accordance with previous research [27]. Cells were then cultured in the complete medium for 42 h, and treated as described above. After washing with PBS for 3 times, images were obtained with a confocal microscope (TCS SP8, Leica, Germany).

### Total protein extraction and western blotting

As described in previous research [28], the treated cells or the mammary tissues ground by tissuelyser (SCIENTZ-12, Ningbo, China) were lysed with RIPA containing 1 mmol/L phenylmethylsulfonyl fluoride (PMSF) and 1 mmol/L protein phosphatase inhibitor. After centrifuging at 13,000 × *g* for 10 min, the liquid supernatant was collected and a Protein Quantification Kit (KTD3001, Abbkine, China) was applied to determine the protein concentration.

20 µg protein samples per lane were loaded onto 12% SDS-PAGE (EC0004, SparkJade, China), sperated, and transferred onto PVDF membranes (IPVH00010, MerckMillipore, USA). The membranes were then incubated with 5% BSA at room temperature for 1 h and primary antibodies at 4 °C overnight successively. The following primary antibodies were used: cGAS (1:1,000, A23846, ABclonal); STING (1:1,000, bs-8335R, Bioss); TBK1 (1:1,000, 28397-1-AP, Proteintech); p-TBK1 (1:1,000, AP1026, ABclonal); p65 (1:1,000, 10745-1-AP, Proteintech); p-p65 (1:5,000, AP-124, ABclonal); IκB (1:1,000, 10268-1-AP, Proteintech); p-IκB (1:500, 39A1413, Thermo Fisher); β-actin (1:5,000, 20536-1-AP, Proteintech). Subsequently, membranes were incubated with the HRP conjugated goat anti-rabbit IgG antibody (1:5,000, CW0103S, CWBIO) or HRP conjugated goat anti-mouse IgG antibody (1:5,000, CW0102S, CWBIO) for 1 h. Lastly, the ECL was applied to develop signals, which were observed with a protein imager. The protein expression abundance was assessed by ImageJ software using β-actin as the internal reference.

### Quantitative reverse-transcription PCR analysis

AIPzol total RNA extraction reagent (RE205-02, i-presci, China) was used to isolate total RNA following the product manual. The RNA concentration and the OD_260_/OD_280_ ratio of each sample was assessed by a Thermo Scientific NanoDrop (Thermo Fisher Scientific, USA). Each sample was uniformly diluted into 250 ng/μL and reverse-transcribed into cDNA with the HiScript III 1^st^ Strand cDNA Synthesis Kit (+gDNA wiper) (R323, Vazyme, China). The cocktail for reverse-transcriptional product, Taq Pro Universal SYBR qPCR Master Mix (Q712, Vazyme, China) and specific primers for target genes were mixed and combined with DEPC water to 20 μL. Reactions were then detected with the 7500 real-time PCR system (Thermo Fisher Scientific, USA). The relevant primer sequences are presented in Table S3. The relative mRNA abundance was calculated using the 2^–ΔΔCT^ method based on the cycle threshold using β-actin as the internal control.

### Statistical analysis

Data were collected from at least three separate trials and analyzed with SPSS Statistics 26.0 (IBM Corp., Armonk, NY, USA) and GraphPad Prism 9 (GraphPad Software, San Diego, CA, USA). Shapiro–Wilk test and Levene’s test were performed to verify the normality and homogeneity of variances, respectively. For the comparison between healthy and ketotic cows, as well as multiple-group comparisons, data that met the assumption of a Gaussian distribution were analyzed using an independent samples *t*-test and a one-way ANOVA followed by Tukey’s post hoc test. Data are expressed as the mean ± SD (standard deviation) or the median and IQR (interquartile range). *P*-value < 0.05 was considered statistically significant.

## Results

### Baseline characteristics and blood parameters

As shown in Table 1, clinical ketotic cows had higher serum BHB (*P* < 0.05) and NEFA (*P* < 0.05) concentration but lower serum glucose (*P* < 0.05) levels and milk yield (*P* < 0.05). Compared to the control, the clinically ketotic cows had a higher BCS (*P* < 0.05). These results demonstrated that clinical ketotic cows undergo severe negative energy balance.

**Table 1:** Baseline characteristics of cows^1^

Item	Health (*n* = 10)		Ketosis (*n* = 10)		*P*-value^2^
	Median	IQR	Median	IQR	
Number of parities	3.5	3.0, 4.0	3.4	3.0, 4.0	0.754
Body condition scores	3.30	3.06, 3.50	3.68	3.56, 3.75	0.003
Milk yield, kg of milk/cow/d	45.76	42.95, 44.83	41.01	39.05, 43.5	0.039
Serum BHB, mmol/L	0.56	0.50, 0.6	3.68	3.33, 4.05	< 0.001
Serum NEFA, mmol/L	0.13	0.05, 0.12	0.59	0.31, 0.61	0.009
Serum glucose, mmol/L	3.34	3.25, 3.42	2.14	1.66, 2.53	< 0.001

^1^Concentration of serum BHB, NEFA, and glucose in Health (*n* = 10) and Ketosis (*n* = 10) groups are shown as median (interquartile range, IQR)

^2^*P* < 0.05 was considered as significance

### cGAS-STING pathway activation and dsDNA release in cows with clinical ketosis

**Fig. 1:**
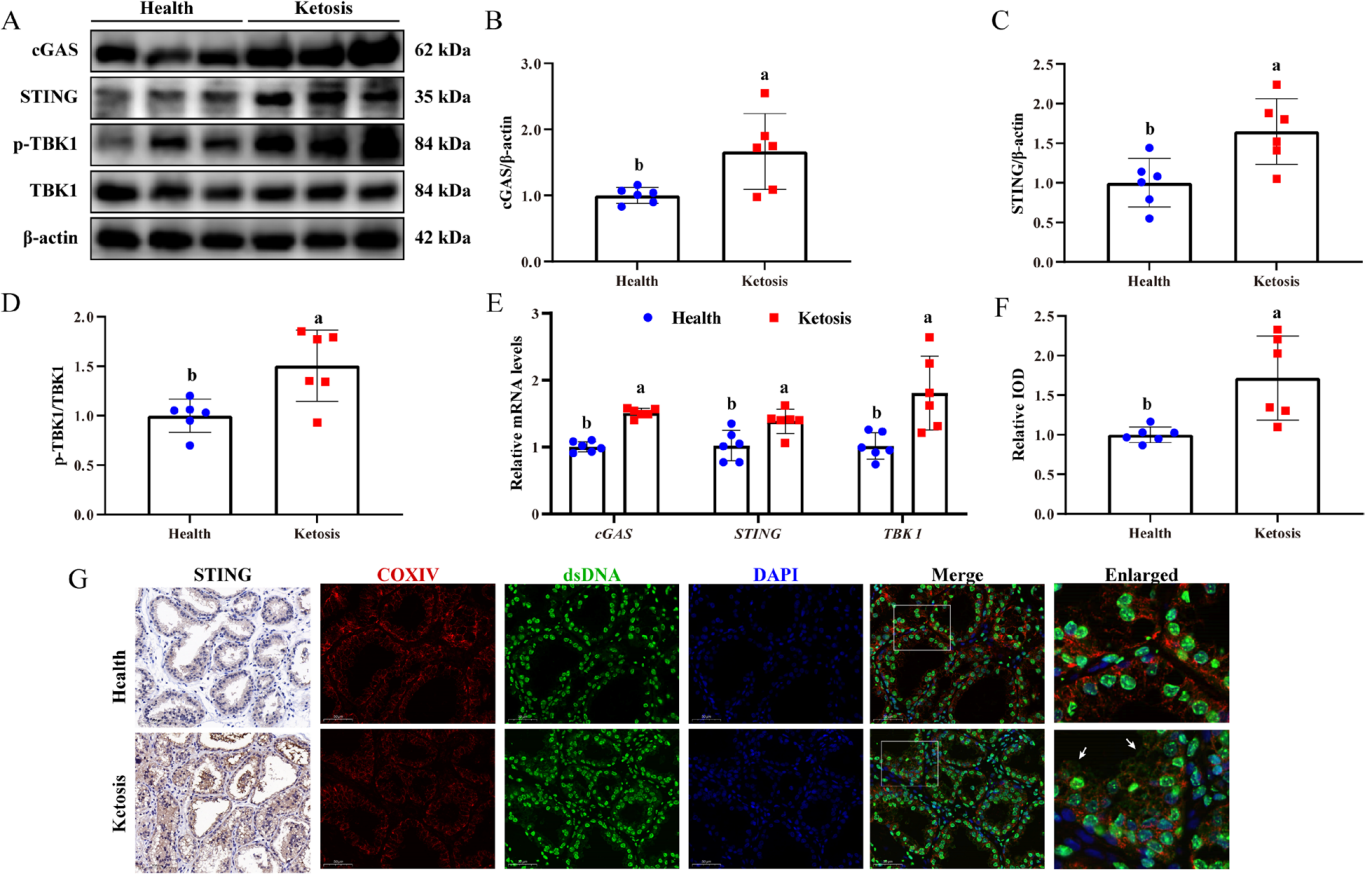
cGAS-STING pathway activation in cows with clinical ketosis. **A** Western blotting for cGAS, STING, p-TBK1, and TBK1 in BHB-treated MAC-T. **B**–**D** Relative protein levels of cGAS, STING, and p-TBK1/TBK1. **E** Relative mRNA levels of *cGAS*, *STING*, and *TBK1*. **F** Relative IOD of STING. **G** IHC staining of STING (Scale bars: 100 µm) and the colocalization of mitochondria (red) and dsDNA (green) in mammary gland tissue (Scale bars: 50 µm). White arrows indicated the dsDNA within the cytoplasm. Data are expressed as the mean ± SD (*n* = 6). Different letters, determined using an independent samples *t*-test, indicate significant differences (*P* < 0.05) relative to the Health group

In comparison to healthy cows, ketotic cows exhibited greater protein levels of cGAS and STING, along with a higher ratio of p-TBK1/TBK1 (Fig. 1A–D, *P* < 0.05). Meanwhile, mRNA levels of cGAS, STING, and TBK1 were observed to be elevated in the mammary gland tissues of clinical ketotic cows (Fig. 1E, *P*< 0.05). The results of IHC staining also showed that the STING was up-regulated in the mammary gland from cows with ketosis (Fig. 1F and G, *P* < 0.05). Consistent with the previous study [8], a higher ratio of p-P65/P65 and p-IκB/IκB as well as the overexpression of inflammatory factors such as interleukin 1 beta (*IL-1B*), interleukin 6 (*IL-6*), and tumor necrosis factor alpha (*TNF-α*) were observed in the cow with ketosis (Fig. S1). These findings showed that cGAS-STING pathway and inflammatory response were activated in the mammary tissue of ketosis cows.

Immunofluorescence co-staining of mitochondria (COXIV) and dsDNA revealed that the abundance of dsDNA (not localized with mitochondria and nucleus) was elevated in the ketosis group (white arrows).

### BHB exposure induced inflammation activation in MAC-T

**Fig. 2:**
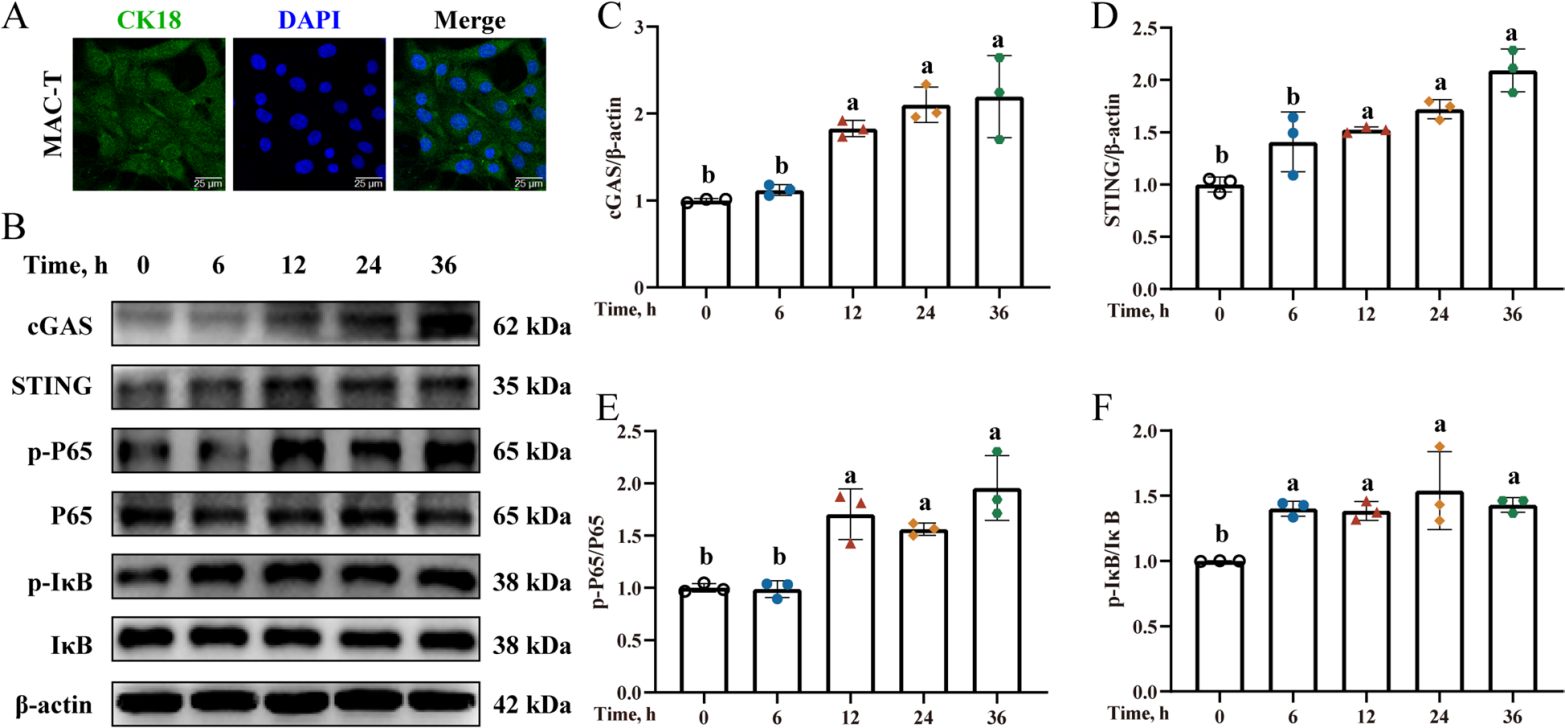
BHB exposure induce inflammatory activation in MAC-T. **A** Typical image of MAC-T. Scale bars: 25 µm. **B** Western blotting for cGAS, STING, p-P65, P65, p-IκB, and IκB in BHB-treated MAC-T. **C**–**F** Relative protein levels of cGAS, STING, p-P65/P65, and p-IκB/IκB. MAC-T were treated with 2.4 mmol/L BHB for 0, 6, 12, 24, and 36 h. Data are expressed as the mean ± SD (*n* = 3). Different letters, determined using one-way ANOVA, indicate significant differences (*P* < 0.05) relative to the 0 h group

MAC-T was applied for the subsequent in vitro experiments (Fig. 2A). As shown in Fig. 2B–D, the protein abundance of cGAS and STING were notably higher in MAC-T after 12, 24, and 36 h of 2.4 mmol/L BHB treatment compared to 0 h (*P* < 0.05). Simultaneously, BHB treatment also enhanced the phosphorylation levels of P65, and IκB (Fig. 2B, E–F, *P* < 0.05). Combining our experimental results with the descriptions of previous studies [23], 24 h was selected as the optimal duration for BHB treatment in the subsequent experiments.

### cGAS-STING pathway activation mediated BHB-induced inflammation in MAC-T

**Fig. 3:**
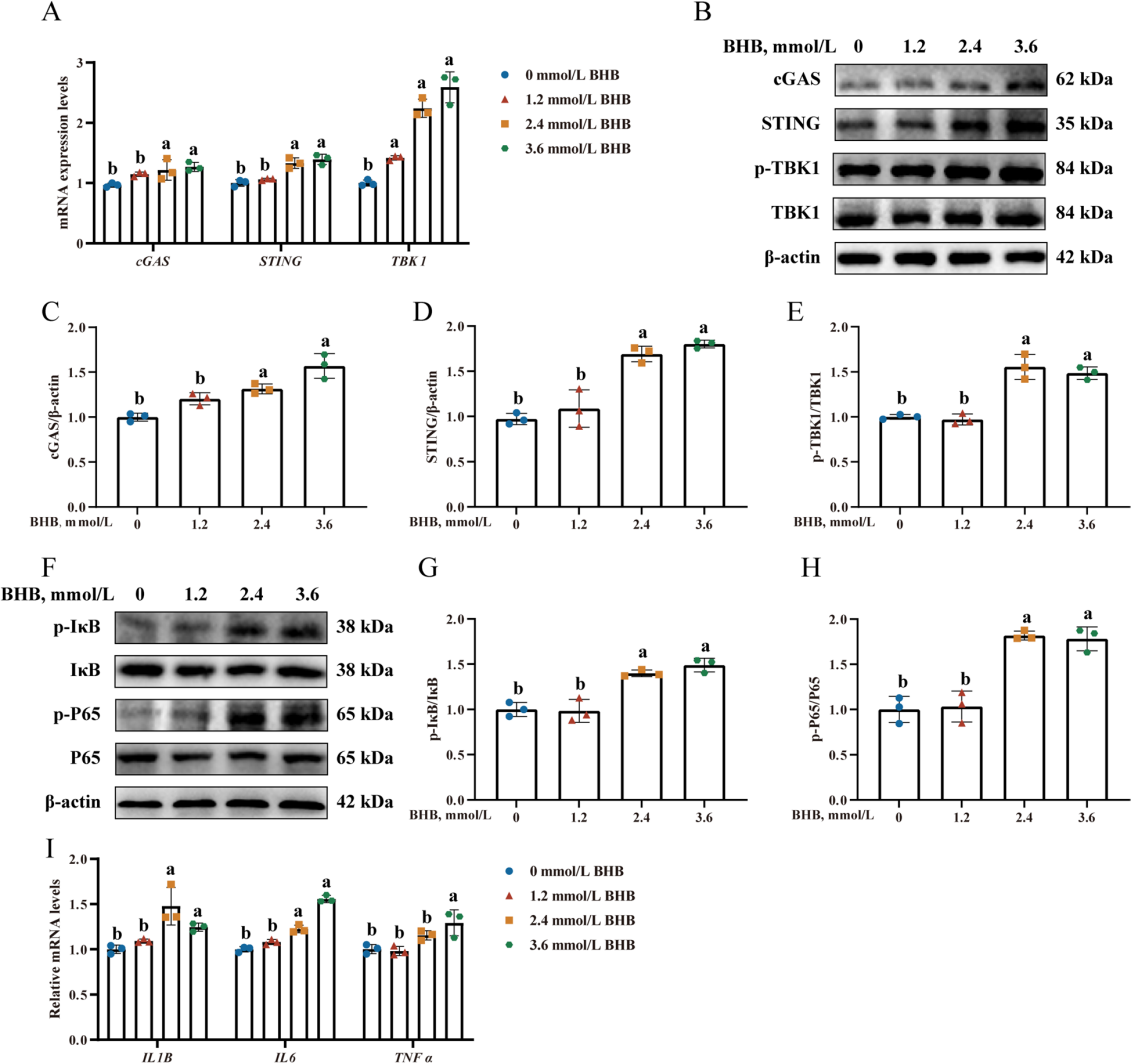
cGAS-STING pathway activation mediated BHB-induced inflammation in MAC-T. **A** Relative mRNA levels of *cGAS*, *STING*, and *TBK1*. **B **Western blotting for cGAS, STING, p-TBK1, and TBK1 in BHB-treated MAC-T. **C**–**E** Relative protein levels of cGAS, STING, and p-TBK1/TBK1. **F **Western blotting for p-P65, P65, p-IκB, and IκB in BHB-treated MAC-T. **G **and **H** Relative protein levels of p-P65/pP65 and p-IκB/IκB. **I** Relative mRNA levels of *IL-1B*, *IL-6*, and *TNF-α*. MAC-T were treated with 0, 1.2, 2.4, and 3.6 mmol/L BHB for 24 h. Data are expressed as the mean ± SD (*n* = 3). Different letters, determined using one-way ANOVA, indicate significant differences (*P* < 0.05) relative to the 0 mmol/L BHB group

In comparison with the 0 mmol/L BHB group, treatment with 2.4 and 3.6 mmol/L BHB up-regulated the transcript abundance of *cGAS*, *STING*, and *TBK1* (Fig. 3A, *P* < 0.05). In addition, the protein levels of cGAS (Fig. 3C, *P* < 0.05) and STING (Fig. 3D, *P*< 0.05), as well as the ratio of p-TBK1/TBK1 (Fig. 3E, *P* < 0.05), increased significantly for 2.4 and 3.6 mmol/L BHB treatment.

The phosphorylation levels of IκB (Fig. 3G, *P* < 0.05) and P65 (Fig. 3H, *P* < 0.05) were higher after treatment with 2.4 and 3.6 mmol/L BHB. In addition, *IL-1B*, *IL-6*, and *TNF-α* were significantly increased (Fig. 3I, *P* < 0.05). Thus, 2.4 mmol/L BHB was chosen as suitable to assess the mechanisms whereby BHB harms mammary cells. Given the above, our results further demonstrate that BHB stress triggers cGAS-STING pathway activation and subsequently inflammation response in MAC-T.

### si-STING transfection effectively alleviated BHB-induced inflammation in MAC-T

**Fig. 4:**
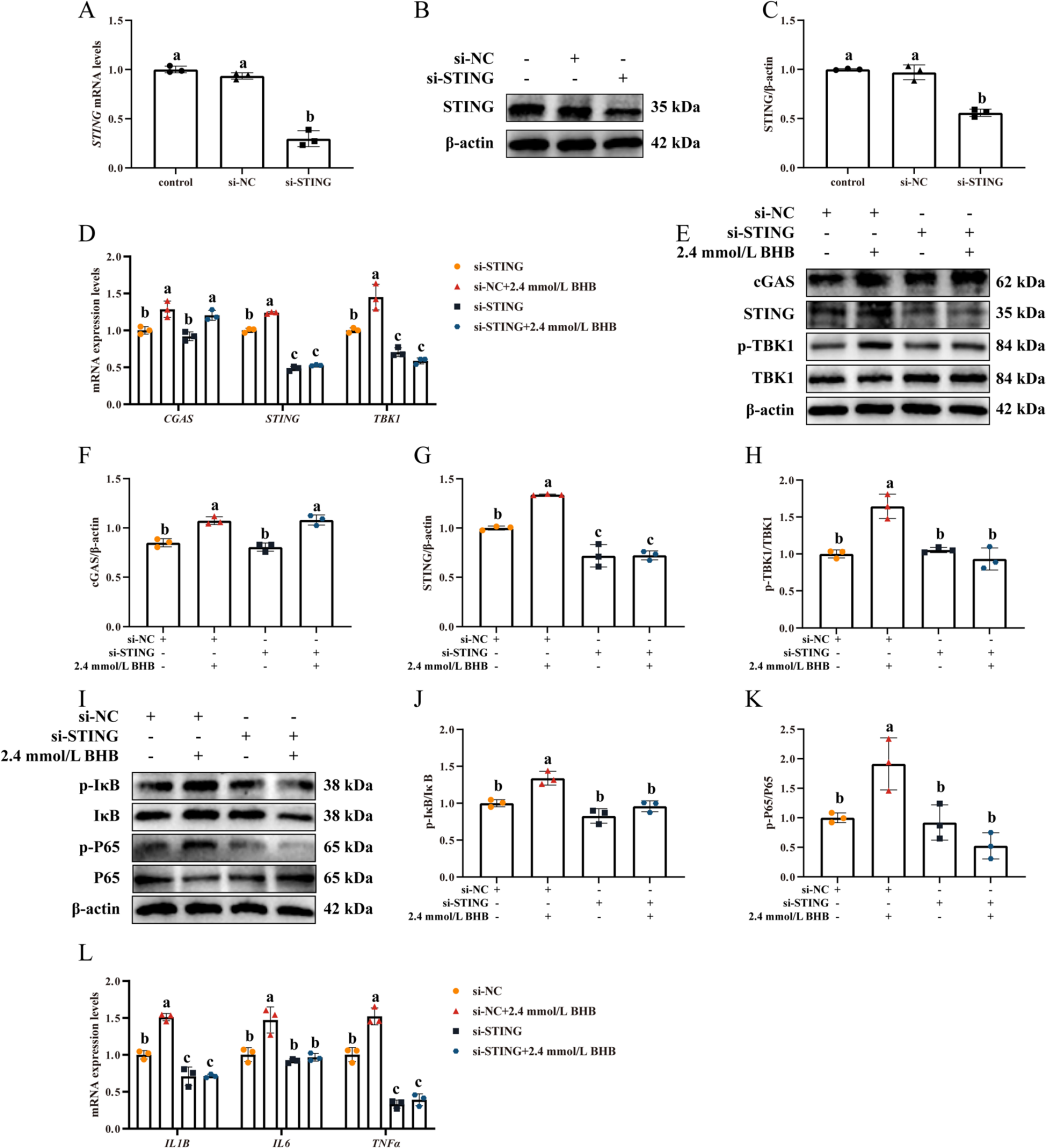
si-STING transfection effectively alleviated BHB-induced inflammation in MAC-T. **A**–**C** Inhibition efficiency of si-STING in MAC-T. **D** Relative mRNA levels of *cGAS*, *STING*, and *TBK1*. **E **Western blotting for cGAS, STING, p-TBK1, and TBK1 in MAC-T. **F**–**H** Relative protein levels of cGAS, STING, and p-TBK1/TBK1. **I** Western blotting for p-P65, P65, p-IκB, and IκB in MAC-T. **J**–**K** Relative protein levels of p-P65/P65 and p-IκB/IκB. **L** Relative mRNA levels of *IL-1B*, *IL-6*, and *TNF-α*. After si-NC or si-STING transfection, MAC-T were treated with 0 and 2.4 mmol/L BHB for 24 h. Data are expressed as the mean ± SD (*n* = 3). Different letters, determined using one-way ANOVA, indicate significant differences (*P* < 0.05) between groups

To further explore the implication of the cGAS-STING signaling in BHB-mediated effect on inflammation in MAC-T, si-STING was used. As in Fig. 4A–C, si-STING transfection effectively reduced the transcription and translation levels of STING in MAC-T (*P* < 0.05), with no significant difference observed between the si-NC and the control groups.

The mRNA levels of *STING* (*P* < 0.05), and *TBK1* (*P* < 0.05) were attenuated in response to BHB treatment with si-STING transfection (Fig. 4D). Compared with the si-NC + 2.4 mmol/L BHB group, the protein levels of STING (Fig. 4G, *P* < 0.05) and p-TBK1/TBK1 (Fig. 4H, *P* < 0.05) were downregulated in the si-STING + 2.4 mmol/L BHB group, but there was no significant difference in the cGAS mRNA and protein abundance (Fig. 4D and F, *P* > 0.05). Meanwhile, the levels of p-IκB/IκB (Fig. 4J, *P* < 0.05) and p-P65/P65 (Fig. 4K, *P* < 0.05) were significantly decreased in the si-STING + 2.4 mmol/L BHB group. The relative mRNA levels of *IL-1B*, *IL-6*, and *TNF-α* also has a similar tendency (Fig. 4L, *P* < 0.05).

### BHB exposure induced autophagic defect and mtDNA release in MAC-T

**Fig. 5:**
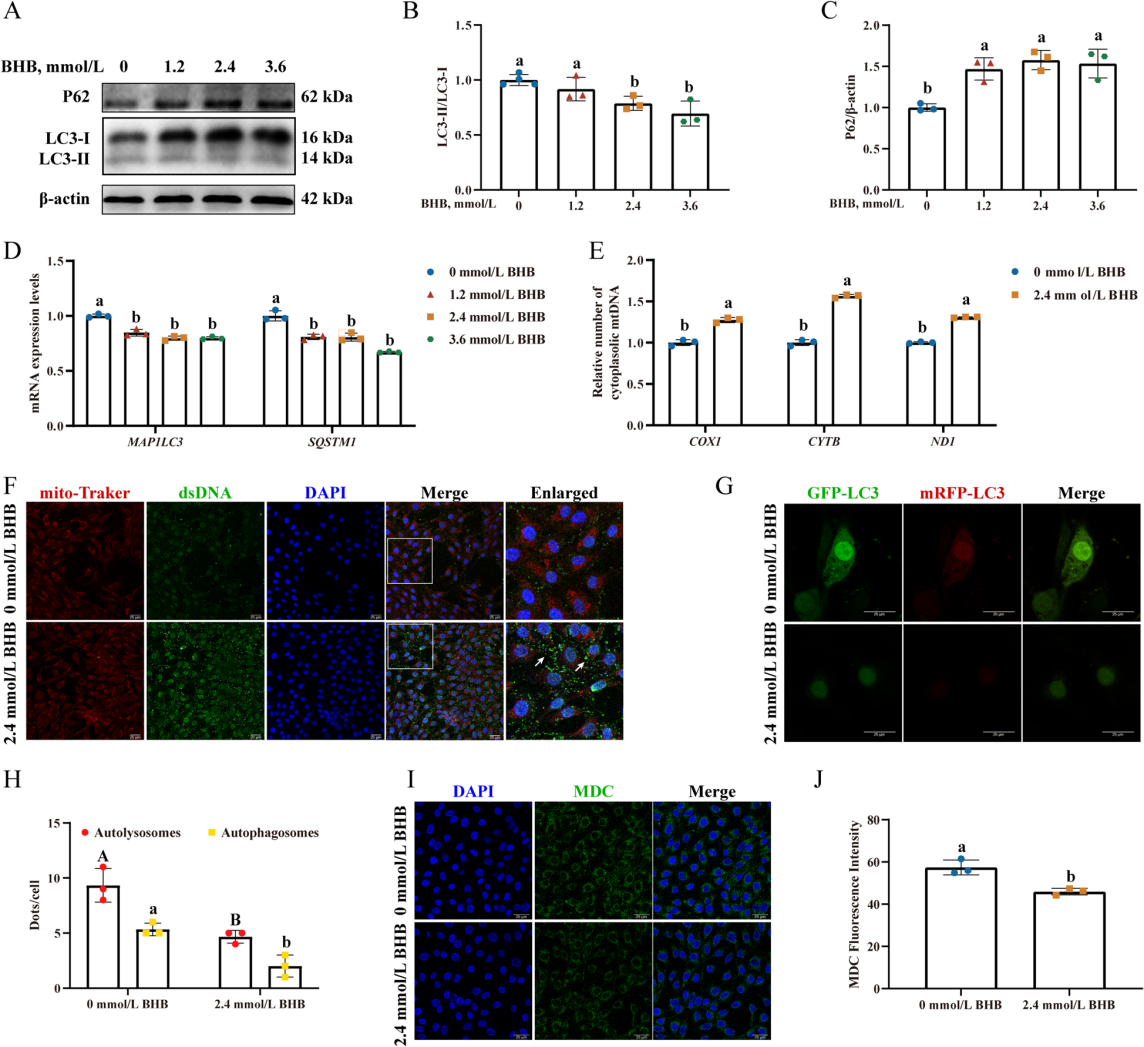
BHB exposure induced autophagic defect and mtDNA released in MAC-T. **A **Western blotting for P62, LC3-II, and LC3-I in MAC-T. **B** and **C** Relative protein levels of P62, and LC3-II/LC3-I. **D** Relative mRNA levels of *MAP1LC3*, and *SQSTM1*. **E** Relative abundance of cytoplasmic mtDNA in MAC-T. **F** The colocalization of mitochondria and dsDNA in MAC-T. White arrows indicate the dsDNA within cytoplasm. Scale bars: 25 µm. **G** and **H** Representative images of GFP-LC3 and mRFP-LC3. Red fluorescence represents autolysosomes, and yellow fluorescence indicates autophagosomes (merged red and green). Scale bars: 25 µm. **I** and **J** MDC staining in MAC-T. Scale bars: 25 µm. MAC-T were treated with 0, 1.2, 2.4, and 3.6 mmol/L BHB for 24 h. Data are expressed as the mean ± SD (*n* = 3). Different letters, determined using one-way ANOVA, indicate significant differences (*P* < 0.05) relative to the 0 mmol/L BHB group

2.4 and 3.6 mmol/L BHB treatment significantly decreased the ratio of LC3-II/LC3-I (Fig. 5B, *P *< 0.05) as well as the mRNA levels of *MAP1LC3* (Fig. 5D, *P* < 0.05). Interestingly, BHB treatment notably upregulated the P62 abundance (Fig. 5C, *P* < 0.05) but decreased the mRNA levels of *SQSTM1* (Fig. 5D, *P* < 0.05). A similar tendency was observed in the cows with ketosis (Fig. S2). Additionally, compared with the control, the 2.4 mmol/L BHB group exhibited a reduction in the number of autolysosomes and autophagosomes (Fig. 5G and H, *P*< 0.05), as well as decreased MDC fluorescence intensity (Fig. 5I and J, *P* < 0.05).

As depicted in Fig. 5E, 2.4 mmol/L BHB also markedly increased the relative number of the typical mitochondrial-code genes cytochrome b (*CYTB*), NADH dehydrogenase subunit 1 (*ND1*), and cytochrome C oxidase subunit I (*COXI*) (*P* < 0.05). Intuitively, 2.4 mmol/L BHB exposure induced severe cytoplasmic dsDNA leakage (White arrows) (Fig. 5F). These data demonstrated that BHB stress leads to the release of mtDNA in MAC-T.

### Rapa pretreatment effectively alleviated BHB-induced autophagic defect and mtDNA release

**Fig. 6:**
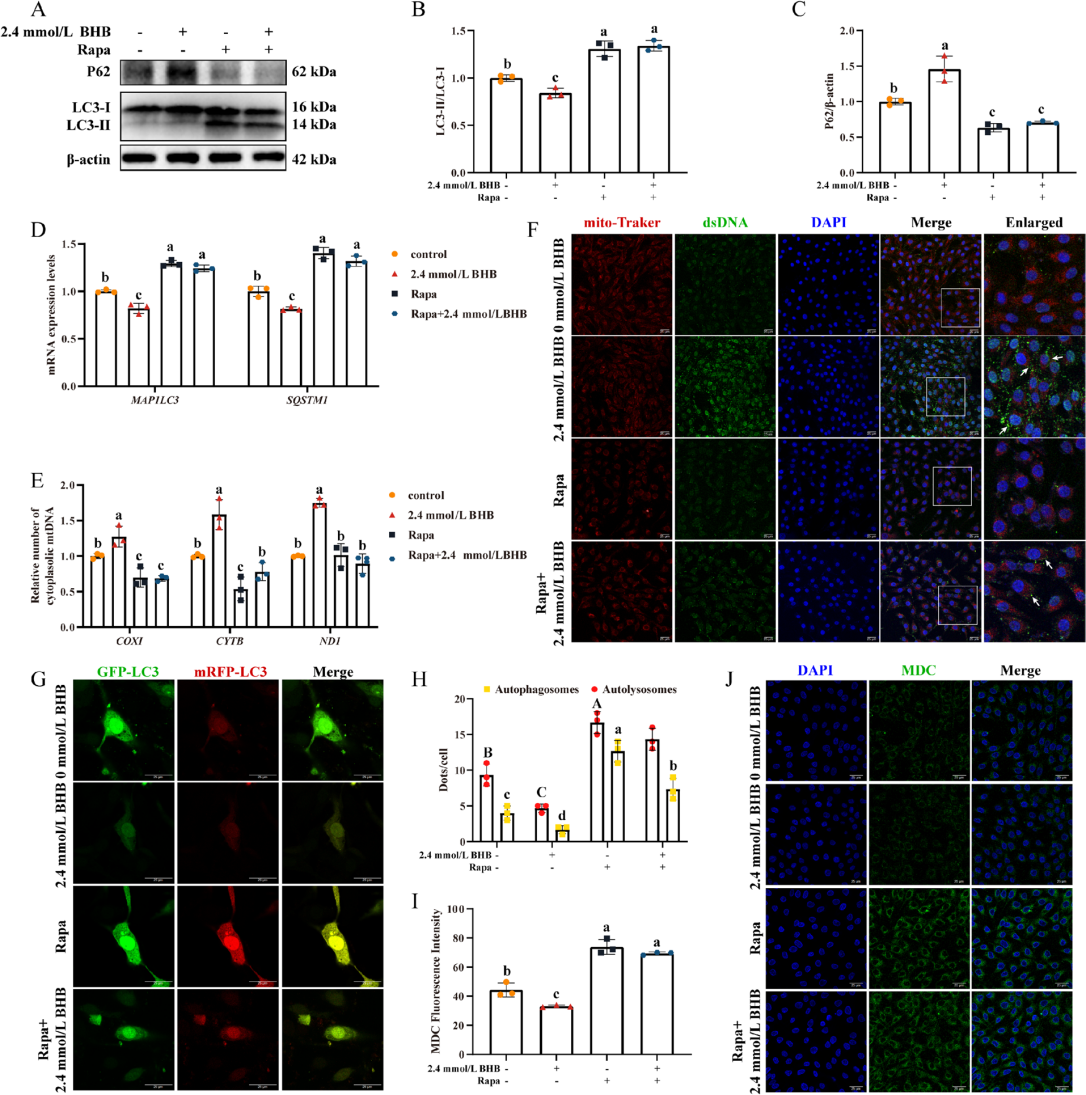
Rapa pretreatment effectively alleviated BHB-induced autophagic defect and mtDNA released in MAC-T. **A** Western blotting for P62, LC3-II, and LC3-I in MAC-T. **B** and **C** Relative protein levels of P62, and LC3-II/LC3-I. **D** Relative mRNA levels of *MAP1LC3*, and *SQSTM1*. **E** Relative abundance of cytoplasmic mtDNA in MAC-T. **F** The colocalization of mitochondria and dsDNA in MAC-T. White arrows indicate the dsDNA within cytoplasm. **G** and **H** Fluorescence intensity of GFP-LC3 and mRFP-LC3. Red fluorescence represents autolysosomes, and yellow fluorescence indicates autophagosomes (merged red and green). **I** and **J** MDC staining in MAC-T. MAC-T were pretreated with or without 100 nmol/L Rapa for 2 h and then stimulated with 0 and 2.4 mmol/L BHB for 24 h. Data are expressed as the mean ± SD (*n* = 3). Different letters, determined using one-way ANOVA, indicate significant differences (*P* < 0.05) between groups

As mentioned above, autophagy was significantly inhibited in MAC-T after BHB treatment. Therefore, we attempted to explore the role of the autophagic defect in BHB-mediated mtDNA released in MAC-T. Compared to the 2.4 mmol/L BHB group, Rapa pretreatment significantly up-regulated LC3-II/LC3-I (Fig. 6B, *P* < 0.05), MAP1LC3 (Fig. 6D, *P* < 0.05), and SQSTM1 (Fig. 6D, *P* < 0.05) but down-regulated the P62 protein levels (Fig. 6C, *P* < 0.05). In addition, Rapa pretreatment also enhanced the number of autolysosomes and autophagosomes (Fig. 6G and H, *P* < 0.05) and the MDC fluorescence intensity (Fig. 6I and J, *P* < 0.05), indicating an improvement in autophagy flux under BHB stress.

In response to BHB treatment, Rapa pretreatment effectively reduced the relative number of *COX*I, *CYTB*, and *ND1* in the cytoplasm (Fig. 6E, *P* < 0.05) and diminished cytoplasmic mtDNA leakage (White arrows) (Fig. 6F).

### Autophagic activation alleviated BHB induced innate inflammation response

**Fig. 7:**
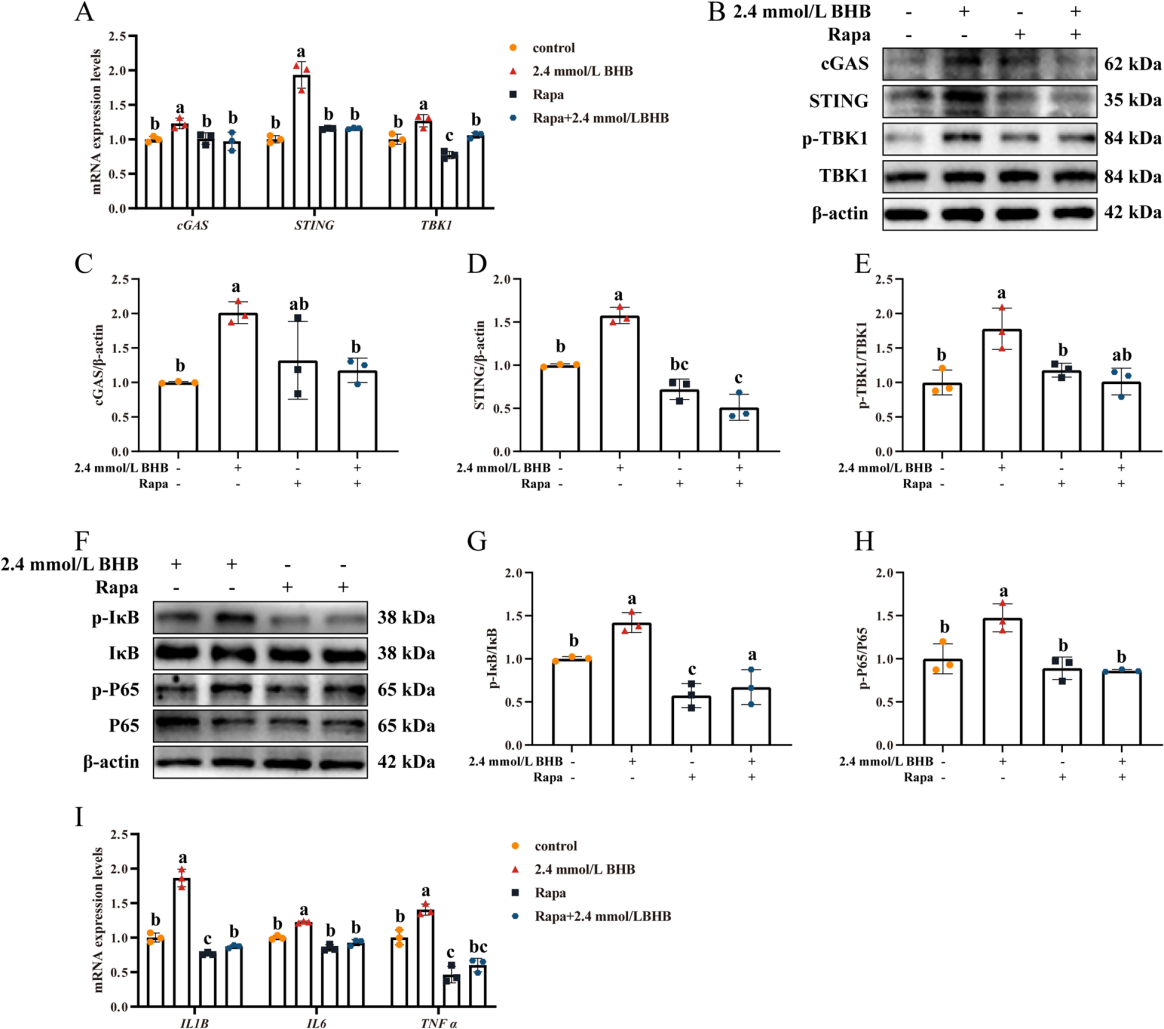
Autophagic activation alleviated BHB induced mtDNA released and inflammation response. **A** Relative mRNA levels of *cGAS*, *STING*, and *TBK1*. **B **Western blotting for cGAS, STING, p-TBK1, and TBK1 in MAC-T. **C**–**E** Relative protein levels of cGAS, STING, and p-TBK1/TBK1. **F **Western blotting for p-P65, p65, p-IκB, and IκB in MAC-T. **G** and **H** Relative protein levels of p-P65/P65 and p-IκB/IκB. **I** Relative mRNA levels of *IL-1B*, *IL-6*, and *TNF-α*. MAC-T were pretreated with or without 100 nmol/L Rapa for 2 h and then stimulated with 0 and 2.4 mmol/L BHB for 24 h. Data are expressed as the mean ± SD (*n* = 3). Different letters, determined using one-way ANOVA, indicate significant differences (*P* < 0.05) between groups

As indicated in Fig. 7A, Rapa pretreatment inhibited the upregulation of *cGAS*,* STING*, and *TBK1* mRNA abundance induced by 2.4 mmol/L BHB (*P* < 0.05). Similarly, the protein levels of cGAS, STING, and the p-TBK1/TBK1 ratio demonstrated a consistent decrease under the same treatment (Fig. 7B–F, *P* < 0.05). Additionally, Rapa pretreatment also inhibited the phosphorylation activation of IκB and P65 under BHB stress (Fig. 7F–H, *P* < 0.05). Accordingly, compared with cells treated with BHB alone, lower mRNA levels of *IL-1B*, *IL-6*, and *TNF-α* were observed in the Rapa + 2.4 mmol/L BHB group (Fig. 7I, *P* < 0.05).

## Discussion

Ketosis is a metabolic disorder often associated with mammary gland damage and milk production decrease [29]. During ketosis, elevated circulating BHB has been demonstrated to pose metabolic stress and impair cellular function, but the potential mechanisms and underlying relationships among these phenomena remain largely unknown. In this study, we confirmed that cGAS-STING signaling is activated in the mammary glands of ketotic cows. In vitro, 2.4 mmol/L BHB exposure induced autophagic inhibition, mtDNA release, and inflammatory response activation in MAC-T. Mechanistically, excessive BHB caused an innate immune disorder by triggering the cGAS-STING pathway, a typical cytosolic dsDNA sensing machinery. The fact that si-STING transfection and autophagy agonist pretreatment reversed BHB-induced inflammation (as evidenced by inhibition of p65 and IκB phosphorylation levels and down-regulating the expression of *IL-1B*, *IL-6*, and *TNF-α*) supported the hypothesis that mtDNA release in the mammary glands of ketotic cows leads to an endogenous inflammatory response. These results provide insights into potential therapeutic strategies for alleviating inflammation of the mammary gland as well as improving the quantity and quality of milk produced by cows undergoing ketosis.

BHB has emerged as a regulator of inflammation in mammals [30]. In non-ruminants, ketone bodies such as BHB mitigate the pathological inflammatory response during the progression of obesity, liver injury, and other diseases through various strategies [31, 32]. However, ruminants have strong sensitivity to fluctuations in serum BHB levels, which can lead to systematic damage. It has been demonstrated that the rising ketone bodies in the circulatory system and organs due to negative energy balance cause stress responses and aseptic inflammation in mammary gland [8]. In particular, excessive BHB stress has been shown to trigger inflammatory responses in a diverse range of cell types [7, 22, 33]. Such responses were confirmed here BHB stress enhanced the phosphorylation levels of p65 and IκB, as well as increased the mRNA levels of *IL-1B*, *IL-6*, and *TNF-α* in vivo and in vitro. Our data suggest an internal relationship between mammary inflammatory responses and high BHB stress, which merits further experimentation to determine the potential mechanism.

Under metabolic stress conditions, mitochondria are an important source of endogenous host-derived molecules and play a key role in controlling the sterile inflammatory response [34]. These processes are closely associated with mitochondrial dysfunction and increased mtDNA access to the cytosol [9, 35, 36]. Previous research has proposed a sequential relationship between mitochondrial dysfunction and mammary gland damage leading to impaired productivity under ketosis [11]. Notably, mitochondria integrity damage and oxidative stress have been well-established contributors to chronic subclinical inflammation in the mammary glands of ketotic cows [8]. Consequently, we further investigated the mechanism by which BHB promotes inflammation in the mammary gland with a focus on mtDNA release. As expected, the results of immunofluorescent staining in mammary tissues and MAC-T accompanied with increasing abundance of mtDNA within cytoplasm after BHB treatment support the viewpoint that BHB-induced abnormal cytoplasmic mtDNA release.

The cGAS-STING pathway is able to recognise aberrant DNA within the cytoplasm, which has emerged as a pivotal modulator of chronic inflammation [37]. Within the cytoplasm, cGAS recognises and binds to dsDNA, which activates its catalytic capability to synthesise cyclic GMP-AMP (cGAMP). This process triggers the oligomerization and transportation of STING, thereby facilitating the phosphorylation of TANK-binding kinase 1 (TBK1), a serine/threonine kinase [38]. In non-ruminants, cGAS-STING has been linked to various metabolic disorders [37, 39, 40]. The overexpression of cGAS and STING provokes innate immune dysregulation and pathological processes during diabetes or obesity [40, 41]. In the present study, cGAS and STING were overexpressed, and TBK1 phosphorylation levels were higher in mammary gland tissues of ketotic cows and MAC-T incubated with 2.4 and 3.6 mmol/L of BHB, suggesting activation of cGAS-STING pathway under high BHB conditions. Evidence is mounting that oxidative stress and aseptic inflammatory activation in mammary tissue caused by ketosis may be important factors decreasing milk production [42]. It has been confirmed that phosphorylated-activated TBK1 can subsequently activate the phosphorylation of P65 and facilitate the transcription of proinflammatory cytokines [43]. On the contrary, STING inhibition effectively reduced pathology in a number of inflammatory diseases in humans [44]. Herein, our results indicate that STING silencing effectively reversed the BHB-induced inflammatory response. These evidence jointly demonstrated that high levels of BHB induce cytosolic leakage of mtDNA and activate the cGAS-STING pathway, which might contribute to udder damage in the ketotic cow.

The activation of autophagy and lysosomal function has been linked to the adaptive mechanism in cows suffering from ketosis [45, 46]. Previous research also showed that activating autophagy plays a role in suppressing inflammatory damage by promoting the removal of cytoplasmic dsDNA [47, 48]. However, our study indicated an obvious inhibition in the ratio of LC3-II/LC3-I but P62 accumulation in mammary gland tissues of ketotic cow and BHB-treated MAC-T, which were consistent with previous study [18]. Moreover, the weakened fluorescence intensity of MDC and mRFP-LC3 demonstrated a defect in autophagosome formation under BHB stress, which indicated defective autophagic activity in high BHB situations. At least in nonruminants, reduced autophagic activity strikingly correlates with mtDNA turnover defects and mtDNA damage [49]. Shen et al. [50] pointed out that the differential autophagy state in cows with subclinical and clinical ketosis mediates the disparate hepatic oxidative status. To this end, we tested the role of autophagy activation in BHB-induced inflammatory response through Rapa pretreatment, a type of autophagy activator. As expected, our results revealed that autophagy activation effectively decreased cytoplasmic mtDNA abundance, accompanied by inhibited cGAS-STING pathway and inflammation activation. Compelling evidence has suggested that basal autophagy may prevent mtDNA accumulation in neurons, mitigating the activation of proinflammatory processes that are the main reason for neurodegeneration [51]. Altogether, our findings highlight the potential therapeutic of autophagy activation in high level BHB mediated mtDNA release and innate inflammation reactions in MAC-T.

## Conclusions

Our data provide new insights highlighting that increased cytoplasmic mtDNA triggered by high levels of BHB might be the endogenous inflammatory stimulator through initiating the cGAS-STING pathway in the mammary glands during hyperketonemia. In addition, autophagy activation in MAC-T helps to alleviate BHB-induced inflammation by promoting cytoplasmic mtDNA degradation. This study not only elucidates the potential mechanism by which excess BHB triggers inflammation in the mammary epithelium of dairy cows but also provides new evidence for the therapeutic strategy to activate autophagy to alleviate mammary gland inflammation caused by ketosis.

## Supplementary Information

Additional file 1: Table S1 The composition and nutrients levels in the diet for cow, % (DM basis); Table S2 The sequences of si-STING and si-NC; Table S3 qPCR primers and product size; Fig. S1 Inflammatory response activation in cows with clinical ketosis; Fig. S2 Autophagy state in cows with clinical ketosis.

## Data Availability

The datasets used and/or analysed during the current study are available from the corresponding author on reasonable request.

## Abbreviations

BHB: β-Hydroxybutyric acid
IOD: Integrated option density
MAC-T: Bovine mammary epithelial cell line
mtDNA: Mitochondrial DNA
SD: Standard deviation
