# Supplementary material for: β-Hydroxybutyrate-induced mitochondrial DNA (mtDNA) release mediated innate inflammatory response in bovine mammary epithelial cells by inhibiting autophagy
Source: J Anim Sci Biotechnol. 2025 Feb 1;16:15. doi: 10.1186/s40104-024-01143-z (PMC11786434; doi:10.1186/s40104-024-01143-z)
Supplement: Supplementary file 1 — Additional file 1: Table S1 The composition and nutrients levels in the diet for cow, % (DM basis); Table S2 The sequences of si-STING and si-NC; Table S3 qPCR primers and product size; Fig. S1 Inflammatory response activation in cows with clinical ketosis; Fig. S2 Autophagy state in cows with clinical ketosis. [file 40104_2024_1143_MOESM1_ESM.docx]

**Supplementary material**

**Supplementary table**

**Table S1** The composition and nutrients levels in the diet for cow (DM basis, %)

| **Ingredient composition** | **Content** | **Chemical composition** | **Content** |
| --- | --- | --- | --- |
| Leymus chinensis, kg | 1.47 | DM, % of TMR | 49.3 |
| Wheat straw, kg | 0.00 | Ash, % | 8.35 |
| New silage, kg | 66.01 | CP, % | 16.55 |
| Corn, kg | 9.05 | NDF, % | 32.19 |
| Corn germ meal, kg | 4.89 | NFC, % | 42.09 |
| Soybean meal, kg | 2.69 | Crude fat, % | 3.38 |
| Rapeseed meal, kg | 3.67 | Calcium, % | 0.90 |
| Cottonseed, kg | 3.67 | Phosphorus, % | 0.47 |
| DDGD, kg | 3.67 | Sodium, % | 0.49 |
| Premix^1^, kg | 4.88 | Potassium, % | 1.11 |
| Total, kg | 100 | Chlorine, % | 0.54 |
|  |  | Sulfur, % | 0.23 |
|  |  | Magnesium, % | 0.40 |
|  |  | DCAM^2^, meq/100g | 20.17 |
|  |  | ME, Mcal/kg | 2.65 |
|  |  | NE_L_, Mcal/kg | 1.71 |
|  |  | NEm, Mcal/kg | 1.71 |
|  |  | NEg, Mcal/kg | 1.12 |

DDGS = distillers dried grains with solubles; TMR = total mixed ration; DM = dry matter; CP = crude protein; NDF = neutral detergent fiber; NFC = non-fiber carbohydrates; DCAD = dietary anion cation difference; ME = metabolizable energy; NE_L_ = net energy for lactation; NEm = energy for maintainance; NEg = net energy for gain.

^1^Premix contained the following ingredients per kilogram of diet: Crude protein, 250g; Crude Fiber, 120g; Crude Ash, 450g; Ca (HCO3)_2_, 70g; NaCl, 30g; Lysine, 2g; Total phosphorus, 3g; Mn, 33.59 mg; Zn, 35.59 mg; and Cu, 8.55 mg.
^2^DCAD = (Na^+^ + K^+^) - (Cl^-^ + S^2-^)

**Table S2** The sequences of si-STING and si-NC

|  | **Sense sequences (5′-3′)** | **Antisense sequences (5´-3´)** |
| --- | --- | --- |
| si-STING | CCCGGAUCCAAAUUUACAATT | UUGUAAAUUUGGAUCCGGGTT |
| Si-NC | UUCUCCGAACGUGUCACGUTT | ACGUGACACGUUCGGAGAATT |

**Table S3** qPCR primers and product size

| **Gene** | **Gene Bank** | **Primer sequences (5´-3´)** | **PCR product size, bp** | |
| --- | --- | --- | --- | --- |
| *cGAS* | XM_024996918.1 | 5'- AGGAGATATCCGTAGCGGC-3'  5'- TCATTAGGAGCAGAAATCTTCACT-3' | 148 |  |
| *STING* | NM_001046357.2 | 5'- GTGCAGTGTGTATGCTTGGC-3'  5'- GGCTGGAGTGAGGCATCTTC-3' | 140 |  |
| *TBK1* | XM_005206636.4 | 5'- TGCAGCTACTGGATCACTGC-3'  5'- AGCAGAACTTGAAGACCCCG-3' | 121 |  |
| *IL1B* | NM_174093.1 | 5'- CTGAACCCATCAACGAAA-3'  5'- ATGACCGACACCACCTGC-3' | 190 |  |
| *IL6* | NM_173923.2 | 5'- ACAGCTATGAACTCCCGCTT -3'  5'- TCGACCATGCGCTTAATGAGA -3' | 191 |  |
| *TNF-α* | XM_005223596.4 | 5'- CCACGTTGTAGCCGACATCA -3'  5'- ATGAGGTAAAGCCCGTCAGC -3' | 132 |  |
| *MAP1LC3* | NM_001001169.1 | 5'- TAAGGAAACCGTGCTGCTGT -3'  5'- GCAGTGGTGTTTTTCCGTGT -3' | 124 |  |
| *SQSTM1* | NM 176641.1 | 5'- GCCCTGACTACGACCTATGC -3'  5'- GGGATCTTCCGATGGACCAG -3' | 263 |  |
| *β-actin* | NM_173979.3 | 5'- CTAACAGTCCGCCTAGAAGCA -3'  5'- GTCATCACCATCGGCAATGAG -3' | 402 |  |
| *CYTB* | NC_006853.1 | 5'- CGAATGAATCTGAGGCG-3'  5'- TGTTGGGTTGTTGGAGC-3' | 145 |  |
| *ND1* | NC_006853.1 | 5'- GAACCACTACGACCCGCTAC -3'  5'- ACGGCTAGGCTTGATATGGC -3' | 164 |  |
| *COX1* | NC_006853.1 | 5'- CTCATTCCTACTACTCCTCGCATCC -3'  5'- GTTGCCTGCTAAGGGAGGGTAC -3' | 85 |  |
| *TERT* | NM_001046242.1 | 5'- GAAACTGCTTGGGAACCACG -3'  5'- ACACCAGGGCACTTCTGATG -3' | 114 |  |

**Supplementary figure**


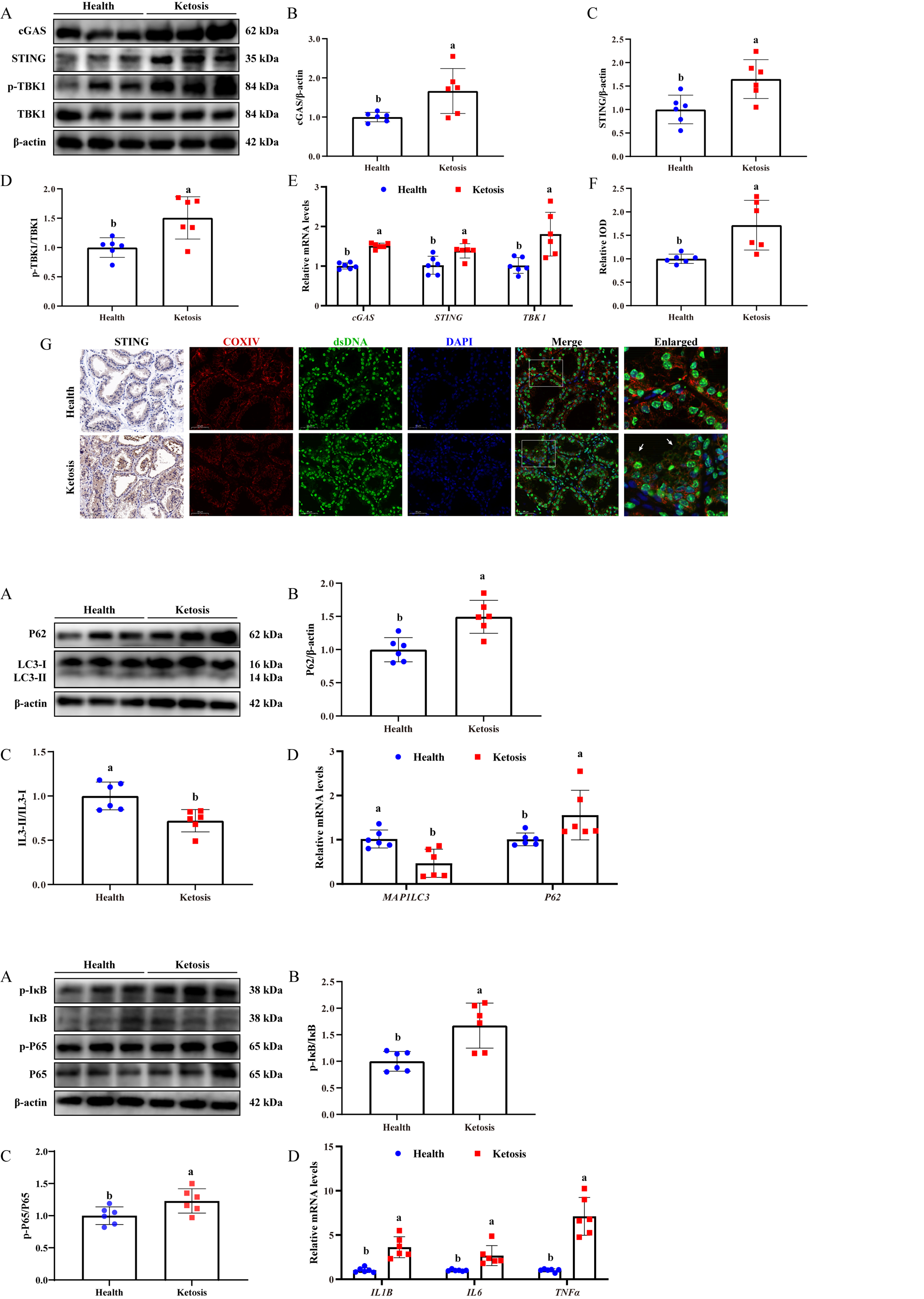


**Fig. S1** Inflammatory response activation in cows with clinical ketosis. **A** Western blotting for p-p65, p65, p-IκB, and IκB in BHB-treated MAC-T. **B** and **C** Relative protein levels of p-P65/P65 and p-IκB/IκB. **D** Relative mRNA levels of *IL-1B*, *IL-6*, and *TNF-α*. Data are expressed as the mean ± SD (*n* = 6). Different letters, determined using an independent samples *t*-test, indicate significant differences (*P* < 0.05) relative to the Health group


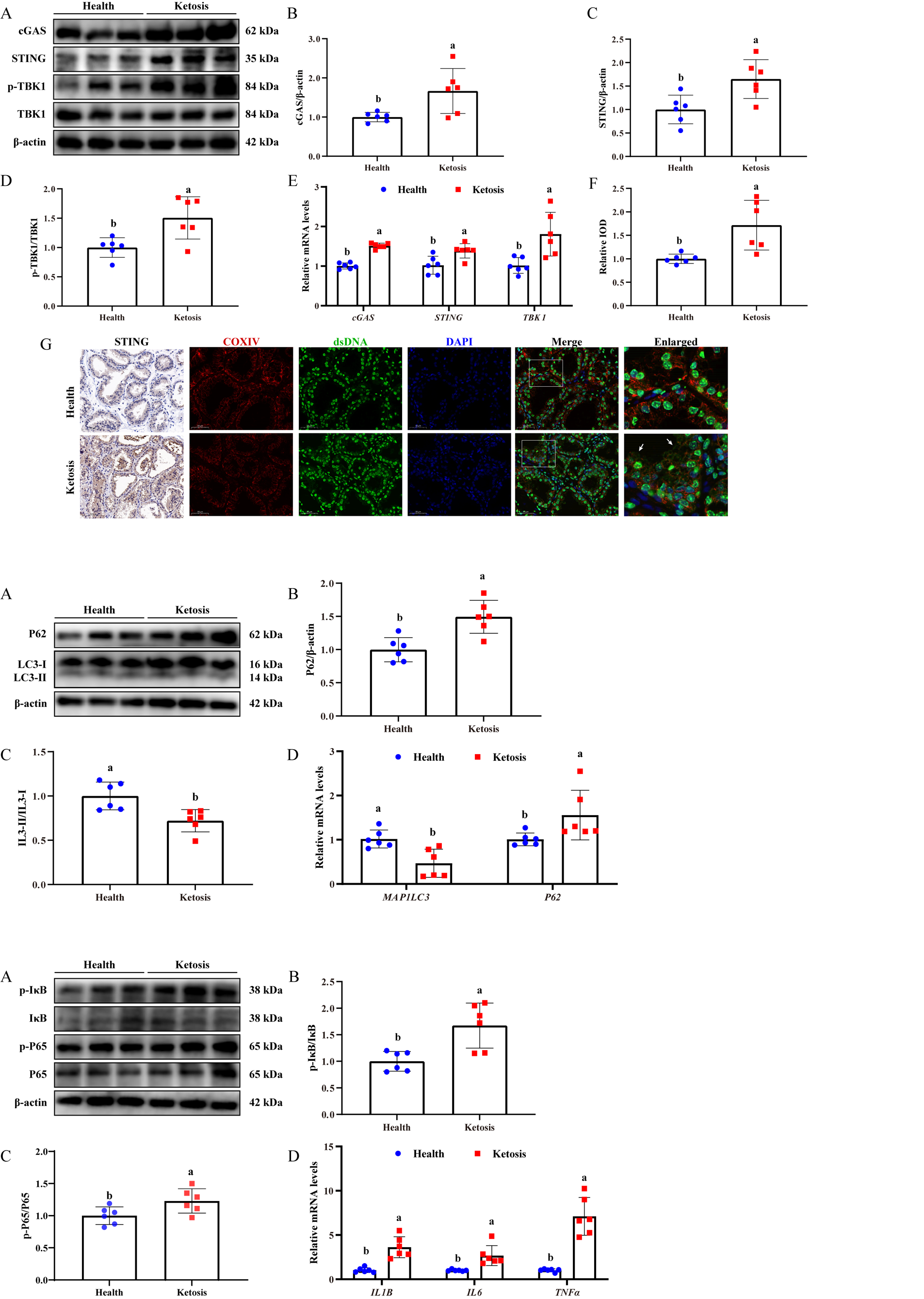


**Fig. S2** Autophagy state in cows with clinical ketosis. **A** Western blotting for P62, LC3-II, and LC3-I in MAC-T. **B** and **C** Relative protein levels of P62, and LC3-II/LC3-I. **D** Relative mRNA levels of *MAP1LC3*, and *SQSTM1*. Data are expressed as the mean ± SD (*n* = 6). Different letters, determined using an independent samples t-test, indicate significant differences (*P* < 0.05) relative to the Health group
